# Biomechanical effects of squatting movements in Tai Chi on the knee joint

**DOI:** 10.3389/fphys.2026.1820135

**Published:** 2026-05-08

**Authors:** Haibin Liu, Wenxiao He, Guang Yang, Suheng Li, Liqing Liu, Fan Gao, Shudong Yan, Ziyang Wang, Fengjie Ran

**Affiliations:** 1 Dalian Third People’s Hospital Affiliated to Dalian University of Technology, Dalian, Liao Ning, China; 2 School of Sports and Health Sciences, Dalian University of Technology, Dalian, Liao Ning, China; 3 Department of Kinesiology and Health Promotion, University of Kentucky, Lexington, KY, United States

**Keywords:** finite element analysis, knee joint biomechanics, sex differences, squat, Tai Chi

## Abstract

**Background:**

Squatting is essential for daily activities but may risk knee injury due to excessive loads. Tai Chi squatting (TCS), characterized by slow, controlled movements, is hypothesized to reduce joint load compared to standard squatting (SS), though biomechanical comparisons remain scarce. This study aimed to biomechanically compare TCS and SS, with a focus on knee joint kinematics, kinetics, muscle activation, and internal stress distribution.

**Methods:**

Twelve experienced Tai Chi practitioners (6 males, 6 females) participated in this study. Participants performed TCS and SS in a controlled laboratory setting. Three-dimensional kinematics were captured with a VICON system, ground reaction forces were measured using AMTI force platforms, and muscle activation was recorded via surface electromyography (sEMG) using the Noraxon Ultium EMG system. The data were processed with an OpenSim musculoskeletal model, and finite element analysis was conducted using Ansys SpaceClaim and Ansys Workbench to evaluate internal knee joint stress distributions.

**Results:**

(1) Kinematics and Kinetics: Compared with SS, TCS produced a markedly smoother knee flexion–extension angle time profile (p < 0.001). TCS elicited significantly greater peak knee extension moments compared with SS (p < 0.001), particularly in male participants, and was associated with significantly elevated activation of the biceps femoris and sartorius, reflecting a shift toward posterior chain co-activation, resulting in a more balanced quadriceps-to-hamstring co-activation pattern. This tendency was particularly pronounced in female participants (p < 0.05). (2) Finite Element Stress Analysis: While the absolute difference in peak Von Mises stress on the femoral cartilage between SS and TCS was modest, TCS produced a qualitatively more homogeneous stress distribution across the articular surface, with the focal high-stress concentration pattern observed under SS notably attenuated. This improvement in load distribution morphology may carry greater functional relevance for long-term cartilage health than peak stress magnitude alone.

**Conclusion:**

TCS promotes more balanced muscle co-activation and a more homogeneous intra-articular stress distribution compared with SS, potentially reducing the risk of localized cartilage fatigue, particularly in female practitioners. These findings support the integration of TCS into rehabilitation and conditioning programs.

## 1 Introduction

The squat is a quintessential closed-chain movement that occupies a central role in activities of daily living, athletic conditioning, and orthopedic rehabilitation. Its execution involves complex musculoskeletal and neuromuscular interactions, during which the knee joint is subjected to substantial mechanical loading (Taketomi et al., 2024; Cotter et al., 2013; Escamilla et al., 2001; Kothurkar et al., 2022). Nevertheless, improper movement mechanics or repetitive high-load squatting have been consistently associated with elevated risk of knee pathology, including osteoarthritis (OA), meniscal tears, and anterior cruciate ligament (ACL) injuries (Felson, 2000; Sharma, 2021). Epidemiological evidence indicates that knee injuries attributable to squatting remain highly prevalent among both occupational populations and competitive athletes (Driban et al., 2020; Allen et al., 2022). Against the backdrop of the Healthy Global initiative, which prioritizes the integration of sport and medicine and advocates for health management across the full life course, identifying training modalities capable of simultaneously enhancing lower-limb function and mitigating joint wear represents a pressing concern in sports biomechanics research (Han & Qiao, 2023; Zhou et al., 2023).

Tai Chi, a distinguished form of traditional Chinese physical exercise, is characterized by slow, controlled, and low-impact movement, with an emphasis on fluidity, continuity, and the harmonious coordination of body and mind (Li et al., 2025; Shao & Song, 2023; Li et al., 2024). As one of the foundational movement patterns within Tai Chi practice, TCS differs substantially from SS. TCS requires practitioners to perform dynamic weight shifting from a markedly lower center of gravity while maintaining strict postural stability (Zhou et al., 2005). In contrast to the load-maximizing objectives of conventional squatting, TCS integrates coordinated breath regulation with continuous center-of-mass displacement, a practice philosophy aimed at enhancing postural control and optimizing joint load distribution (Du et al., 2025). This composite mechanism, which combines controlled descent, dynamic weight transfer, and respiratory coordination, theoretically reduces peak knee joint stress and promotes a more balanced neuromuscular activation pattern. While preliminary evidence suggests that Tai Chi movements may confer articular protective effects relative to conventional exercise (Wang et al., 2022; Wang and Sun, 2022; Li et al., 2023), rigorous quantitative comparisons of the internal knee loading mechanisms between TCS and SS remain scarce. In particular, microscale tissue-level stress evidence derived from FEA is notably absent from the existing literature.

Sex differences constitute another critical determinant of knee injury risk. Owing to anatomical characteristics such as greater quadriceps angle (Q-angle) and wider pelvic geometry, as well as divergent neuromuscular control strategies, females exhibit substantially higher susceptibility to ACL injury and OA than their male counterparts (Koh et al., 2020; Dargel et al., 2009). Sex-specific patterns of muscle activation and lower-limb kinematics during squatting have been documented in the literature (Kreutzinger et al., 2025; Hernandez et al., 2024); however, the extent to which these sex-related differences modulate knee biomechanical responses under the distinctive slow-movement paradigm of Tai Chi remains inadequately understood (Liu L et al., 2024).

In light of these gaps, the present study sought to advance understanding of the biomechanical mechanisms underlying TCS. An integrated methodological framework encompassing three-dimensional motion capture, sEMG, and subject-specific musculoskeletal modeling in OpenSim was employed to quantify knee kinematics and kinetics. A subject-specific finite element model of the knee was subsequently developed to characterize and compare Von Mises stress distributions across articular cartilage and menisci between TCS and SS at the tissue level. The modulatory role of biological sex was further examined as an exploratory analysis. Collectively, these efforts aimed to provide empirical support for the evidence-based application of TCS in knee injury prevention and rehabilitation settings.

The central hypothesis of this study was that, in contrast to SS, which relies predominantly on passive skeletal structures to manage joint loading, TCS exploits enhanced agonist-antagonist co-activation, particularly through augmented recruitment of the posterior chain musculature, to redistribute intra-articular loads. This mechanism was hypothesized to attenuate localized peak stresses on articular cartilage and ligamentous tissues without compromising the overall external training demand. Furthermore, it was anticipated that this protective biomechanical effect would be more pronounced in female participants.

## 2 Methods

### 2.1 Participants

Twelve experienced Tai Chi practitioners with a minimum of five years of continuous training were recruited for this study, comprising six males (age: 41.3 ± 8.8 years; height: 173.7 ± 5.2 cm; body mass: 74.7 ± 8.2 kg) and six females (age: 54.3 ± 3.2 years; height: 162.2 ± 2.8 cm; body mass: 58.3 ± 8.2 kg). All participants were free from musculoskeletal injuries of the lower extremities at the time of testing. Written informed consent was obtained from each participant prior to data collection, and the study protocol was approved by the relevant institutional ethics committee. Before testing commenced, anthropometric parameters including lower limb length, thigh circumference, and ankle circumference were measured and recorded for each participant.

### 2.2 Experimental protocol

Each participant completed two movement conditions in a randomized order under standardized laboratory conditions.

(1) Tai Chi Squat (TCS): The movement adhered to the traditional low-stance (low zhuanbu) requirements of Tai Chi practice. Participants stood with feet shoulder-width apart at 1.5 times the biacromial distance. Squat depth was determined according to the low-stance height formula established by Zhou et al. (2005). Throughout the movement, participants were instructed to embody the Tai Chi principles of upright and relaxed posture (zhongzheng anshu) and fluid, continuous motion (lianguan yuanhuo), maintaining smooth execution and a stable center of mass (Wu et al., 2026; Wu et al., 2004).

(2) Standard Squat (SS): Participants performed a conventional squat with foot placement (1.5 times biacromial width) and squat depth matched to those used in the TCS condition. Participants were instructed to complete a functional lower-limb flexion-extension cycle without incorporating any stylistic elements characteristic of Tai Chi.

Both conditions were performed at a controlled, constant tempo, with each full movement cycle lasting 6 to 8 seconds. A minimum of three trials were completed per condition. The onset of each trial was defined as the moment the participant initiated the descent upon receiving a verbal cue, and trial completion was defined as the moment the participant returned to a stable upright standing posture.

### 2.3 Data collection and processing

**Figure 1:**
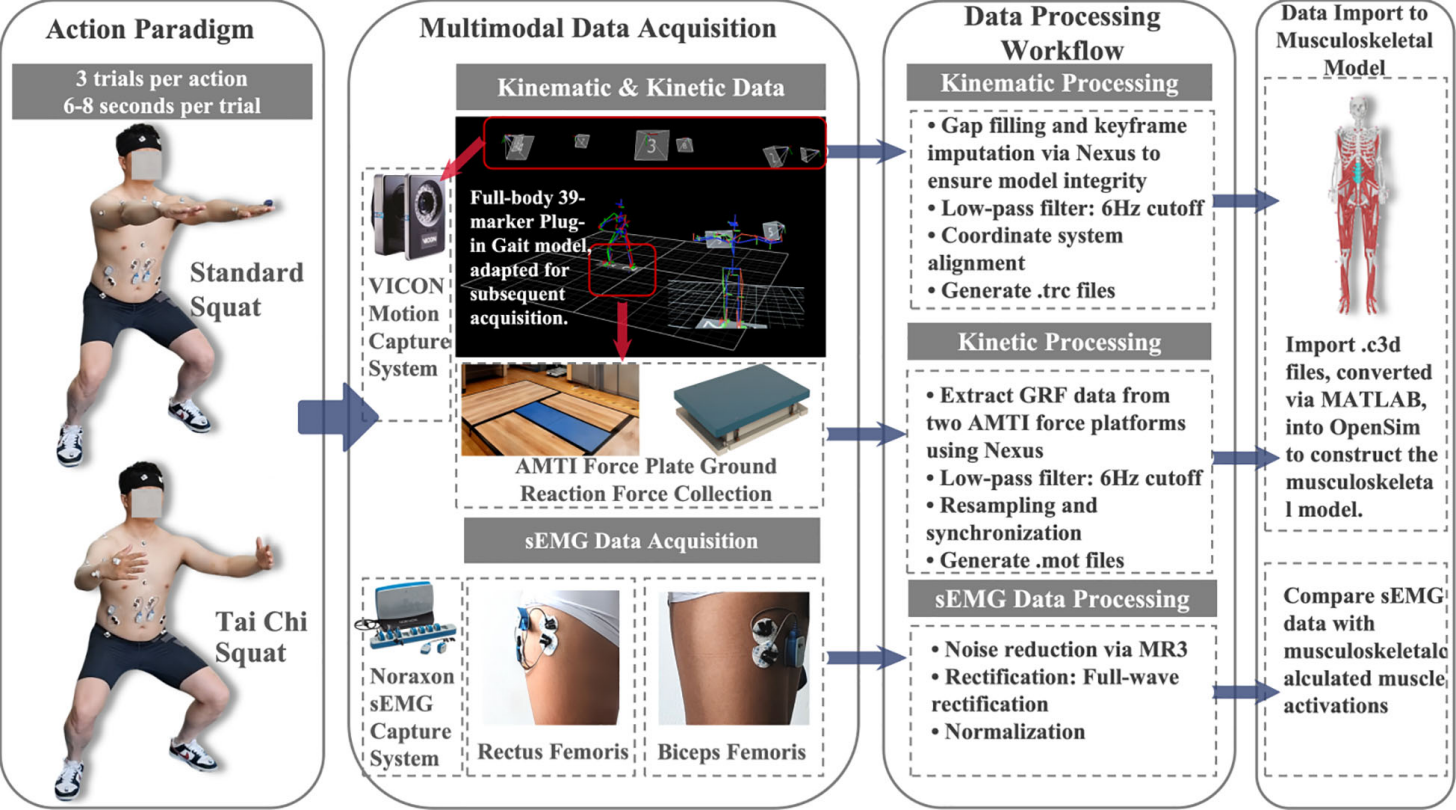
Schematic of experimental protocols, multimodal data acquisition, and preprocessing workflow.

Whole-body kinematic data were collected using an eight-camera three-dimensional motion capture system (Vicon V5, Oxford Metrics, UK) at a sampling frequency of 100 Hz. Retroreflective marker placement followed the Vicon Plug-in Gait full-body model protocol. GRF data were recorded synchronously via two tri-axial force plates (Optima HPS, AMTI, USA) at a sampling frequency of 1,000 Hz. Muscle activation signals from the right rectus femoris (RF) and biceps femoris (BF) were recorded using a wireless sEMG system (Noraxon Ultium, Noraxon, USA) at a sampling frequency of 2,000 Hz. Electrode placement adhered strictly to the SENIAM guidelines. And all kinematic data were low-pass filtered at a cutoff frequency of 6 Hz to attenuate high-frequency noise. Raw sEMG signals were processed sequentially with a 10–500 Hz bandpass filter and a 50 Hz notch filter, followed by full-wave rectification and normalization to peak amplitude. Root mean square (RMS) amplitude was subsequently calculated (Mello et al., 2007). The normalized sEMG envelopes were smoothed using a 50 ms moving average window prior to use in OpenSim model validation. The overall experimental workflow is illustrated in **Figure 1**.

### 2.4 Musculoskeletal modeling and simulation

Subject-specific musculoskeletal models were developed using OpenSim (v4.4, Stanford University, USA). The Full-Body Lumbar Spine (FBLS) model (Rajagopal et al., 2016) was adopted as the base template, comprising 21 rigid body segments, 29 degrees of freedom, and 324 musculotendon units. To align with the experimental marker configuration, the original model was trimmed to retain 39 anatomically defined landmark points (Raabe and Chaudhari, 2016).The simulation pipeline consisted of four sequential steps: (1) Model Scaling, in which model geometry was adjusted to match each participant’s anthropometric dimensions using static calibration data; (2) Inverse Kinematics (IK), to compute time-varying joint angles from the captured marker trajectories; (3) Inverse Dynamics (ID), in which GRF data were combined with segmental kinematics to derive joint moments and joint reaction forces; and (4) Static Optimization (SO), to estimate individual muscle forces. The accuracy of the simulation outputs was validated against the experimentally recorded sEMG data (Seth et al., 2018).

### 2.5 Finite element modeling and analysis

A high-fidelity geometric model of the male knee joint was obtained from the Visible Human Project dataset, funded by the National Institutes of Health (NIH). The selected specimen (age: 39 years; height: 180 cm; body mass: 90 kg) provided anatomically detailed representations of the femur, patella, tibia, articular cartilage, and major ligamentous structures (Andreassen et al., 2023). Geometric reconstruction of the knee joint was performed through a multi-step segmentation workflow. The Visible Human Project dataset was first imported into 3D Slicer (v5.x; slicer.org) for segmentation of bony structures and soft tissue components. A threshold-based semi-automatic segmentation approach was applied, whereby grayscale intensity thresholds were used to isolate each anatomical structure, followed by manual boundary correction on a slice-by-slice basis to ensure geometric accuracy at articular interfaces and regions of low image contrast. The resulting three-dimensional surface geometries were exported as STL files and subsequently imported into Ansys SpaceClaim R1–2022 for mesh generation and pre-processing and subsequently transferred to Ansys Workbench R1–2022 for finite element solution.3 A locally refined meshing strategy was adopted to balance computational efficiency with solution accuracy. Distal regions of the femur and tibia were discretized with a coarser element size of 5 mm, while proximal bone surfaces, articular cartilage, menisci, and all interface regions were meshed with quadratic tetrahedral elements at a finer element size of 1.5 mm to ensure adequate resolution in areas of high stress gradient. This element size is consistent with the mesh convergence findings reported by Kothurkar et al. (2023), in which stress outcomes for cartilage and menisci in a comparable knee FEA model varied by less than 5% when element size was reduced from 1.5 mm to 1.0 mm, confirming the acceptability of 1.5 mm as a converged mesh density for these structures.4

**Figure 2:**
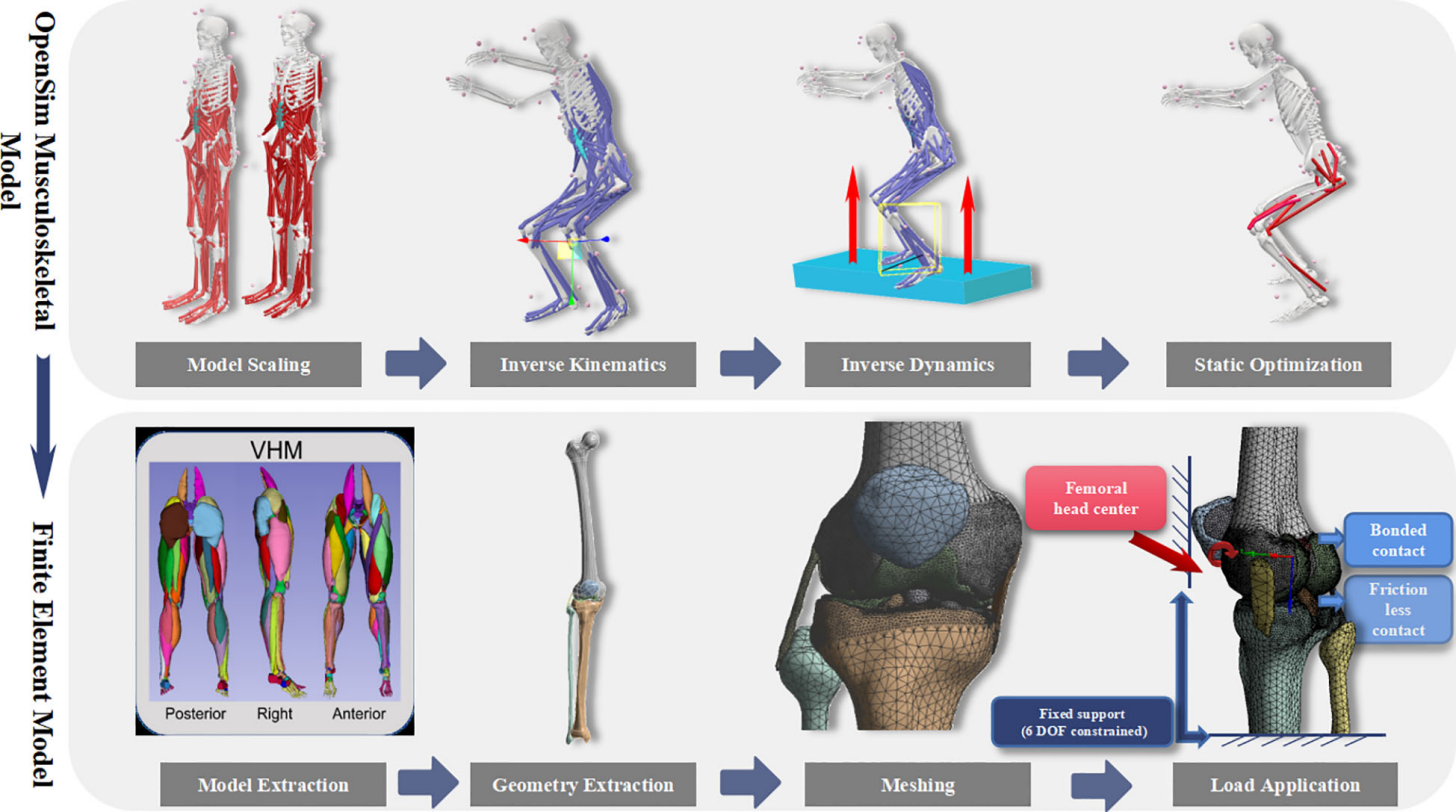
Technical roadmap of coupled musculoskeletal modeling and finite element simulation for the knee joint.

Boundary Conditions and Loading: All six degrees of freedom at the distal ends of the tibia and fibula, as well as at the patellar surface, were fully constrained. Bone-to-cartilage interfaces were defined using bonded contact conditions, while cartilage-to-cartilage interfaces were assigned frictionless contact. All tissues were modeled as isotropic linear elastic materials, with specific material properties listed in **Table 1**. Peak joint moments and muscle forces derived from the OpenSim simulations were applied as dynamic loads at the femoral head center to replicate the intra-articular mechanical environment under each squat condition (Dagneaux et al., 2024; Du et al., 2025). The complete FE modeling workflow is illustrated in **Figure 2**.

**Table 1:** Material properties and mesh details of the knee joint finite element model.

Structure	Material properties	Mesh details
Bones (Femur, Tibia)	Young’s Modulus E = 15000 MPa Poisson’s Ratio v = 0.3	Young’s Modulus E = 15000 MPa Poisson’s Ratio v = 0.3
Cartilage	Young’s Modulus E = 10 MPa Poisson’s Ratio v = 0.45	Tetrahedral elements Element size = 1.5 mm
Ligaments	Young’s Modulus E = 200 MPa Poisson’s Ratio v = 0.3	Tetrahedral elements Element size = 1.5 mm

### 2.6 Data analysis and statistical power

To comprehensively evaluate the biomechanical effects of Tai Chi and standard squatting on the entire lower extremity, this study selected six key muscles for analysis that are critical to the movement and stability of the hip, knee, and ankle joints. These muscles include: gluteus maximus (GLMax), gluteus medius (GM), rectus femoris (RF), biceps femoris (BF), sartorius (SAR), and gastrocnemius (GAS). This selection aimed to capture the complex multi-joint muscle recruitment patterns involved in squatting (Lee et al., 2022; Yuen et al., 2019). All statistical analyses were performed using GraphPad Prism (Version 10.1.2) and Matlab (Version 2023b). Normality of all continuous variables was confirmed via the Shapiro-Wilk test, and data are presented as mean ± standard deviation. A two-way mixed analysis of variance (ANOVA) was employed to examine the main effects of movement condition and sex (between-subject factor: male vs. female), as well as their interaction effect, on knee joint biomechanical outcomes. Where a significant interaction was detected, simple effects analyses were conducted using Sidak or Bonferroni *post hoc* corrections as appropriate. In addition to discrete-parameter comparisons, point-wise t-tests were conducted at each time point across the normalized squat cycle (0–100%) to identify time intervals of statistically significant between-condition differences in all continuous kinematic, kinetic, and muscle activation time-series.6/7 The significance threshold was set at p ≤ 0.05.

## 3 Results

### 3.1 Accuracy verification for results from skeletal muscle simulation

**Figure 3:**
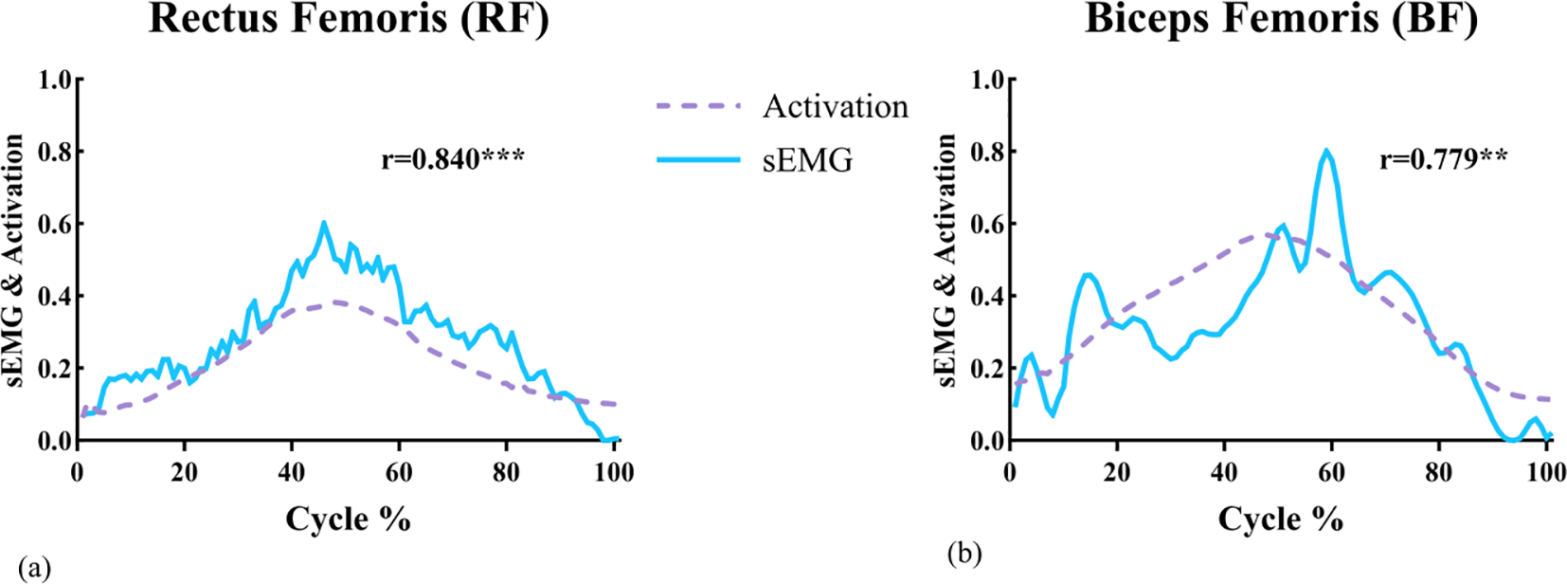
Model validation diagram. Model validation diagram: **(a)** RF and **(b)** BF show the comparison between sEMG signals and muscle activation curves over the squat cycle (%). The range of activation values is from 0 (indicating complete inactivity) to 1 (indicating full activation). ** Indicates: p < 0.01; ***Indicates: p < 0.001.

To ensure the reliability of the simulation outputs, model-derived muscle activation profiles were compared against experimentally recorded sEMG signals. As shown in **Figure 3**, the simulated activation curves for RF and BF demonstrated strong temporal agreement with the experimental data, yielding Pearson correlation coefficients of 0.840 (RF) and 0.779 (BF), respectively. Correlation coefficients exceeding 0.70 are widely recognized as indicative of a strong linear relationship and acceptable model fidelity in musculoskeletal simulation validation contexts (Alexander and Schwameder, 2016; Seth et al., 2018; Raabe and Chaudhari, 2016), and both values obtained in the present study meet this criterion. RF and BF were selected as validation targets as they are the most accessible muscles for reliable surface EMG recording with minimal cross-talk.

### 3.2 Knee joint kinematics and kinetics

**Figure 4:**
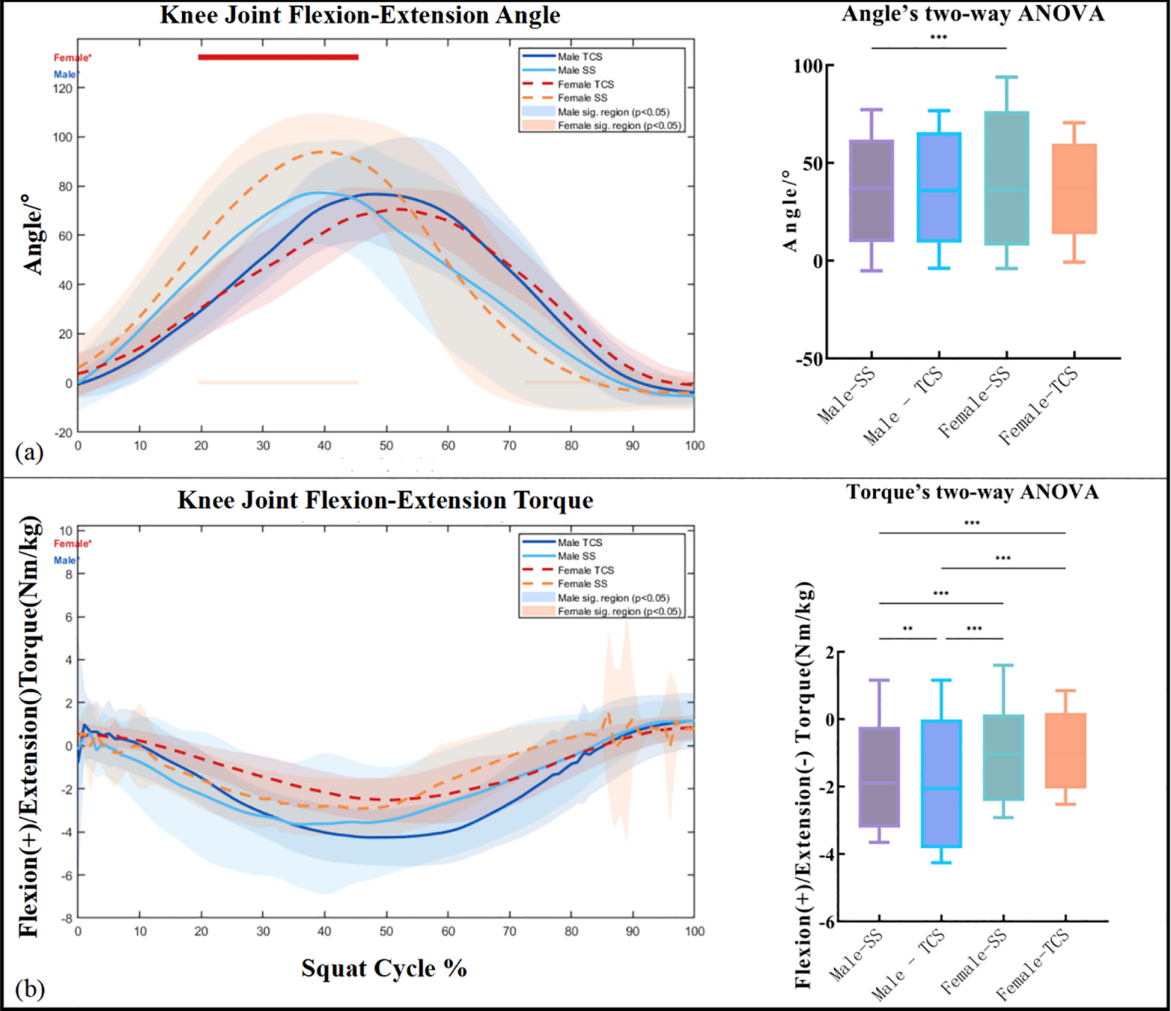
Point-wise and discrete statistical comparisons of knee joint sagittal-plane kinematics and kinetics between TCS and SS. **(a)** Time-series curves of knee joint flexion-extension angle throughout the squat cycle (0–100%) for male (solid lines) and female (dashed lines) participants under TCS and SS conditions, presented as mean ± SD. Shaded bands represent one standard deviation. Horizontal bars above the plot indicate time intervals of statistically significant between-condition differences identified by point-wise t-tests (red: female, p < 0.05; blue: male, p < 0.05). Background color regions correspond to the respective significant time intervals. Right panel: Boxplot comparisons of peak knee flexion angle across the four groups (Male-SS, Male-TCS, Female-SS, Female-TCS), with significance levels from two-way mixed ANOVA indicated by brackets (*p < 0.05, **p < 0.01, ***p < 0.001). **(b)** Time-series curves of knee joint flexion-extension torque (normalized to body mass, Nm/kg) throughout the squat cycle, with the same format as **(a)**. Positive values indicate flexion torque; negative values indicate extension torque. Right panel: Boxplot comparisons of peak torque across the four groups, with ANOVA significance indicated by brackets (**p < 0.01, ***p < 0.001).

**Figure 4A** illustrates the knee joint flexion-extension angle throughout the squat cycle. Both conditions exhibited a characteristic bell-shaped angular trajectory, with peak flexion occurring at approximately 40–50% of the squat cycle. Female participants demonstrated markedly greater peak flexion angles under SS compared with TCS, whereas male participants showed comparatively smaller between-condition differences in angular magnitude. Point-wise comparisons revealed that female participants exhibited significantly greater knee flexion angles during SS relative to TCS across approximately 20–50% of the squat cycle (p < 0.05, red bar in **Figure 4A**), while no sustained significant male-specific interval was identified. Two-way ANOVA confirmed a significant main effect of squat type on peak knee flexion angle (F = 24.87, p < 0.001), with neither the main effect of sex (F = 0.31, p = 0.577) nor the squat type × sex interaction (F = 1.13, p = 0.289) reaching significance.

**Figure 4B** presents the knee joint flexion-extension torque throughout the squat cycle. The y-axis convention defines flexion as positive and extension as negative. Male participants generated substantially larger peak extension torques than female participants under both conditions. Importantly, Male-TCS produced markedly larger peak extension torques than Male-SS, while female participants showed a comparatively smaller between-condition difference. Point-wise comparisons identified significant between-condition torque differences for male participants during the early loading phase (approximately 0–20% of the cycle) and for female participants across a broader mid-cycle interval (p < 0.05, **Figure 4B**). Two-way ANOVA revealed significant main effects of both squat type (p < 0.001) and sex (p < 0.001), along with a significant squat type × sex interaction (p < 0.001), confirming that the torque response to TCS was modulated differently between sexes. These findings indicate that, despite its slow and controlled movement characteristics, TCS imposes greater knee extension moment demands than SS, particularly in male participants, consistent with the elevated antagonist co-activation requirements of the Tai Chi movement paradigm.

### 3.3 Effect of gender and squat type on muscle activation patterns

**Figure 5:**
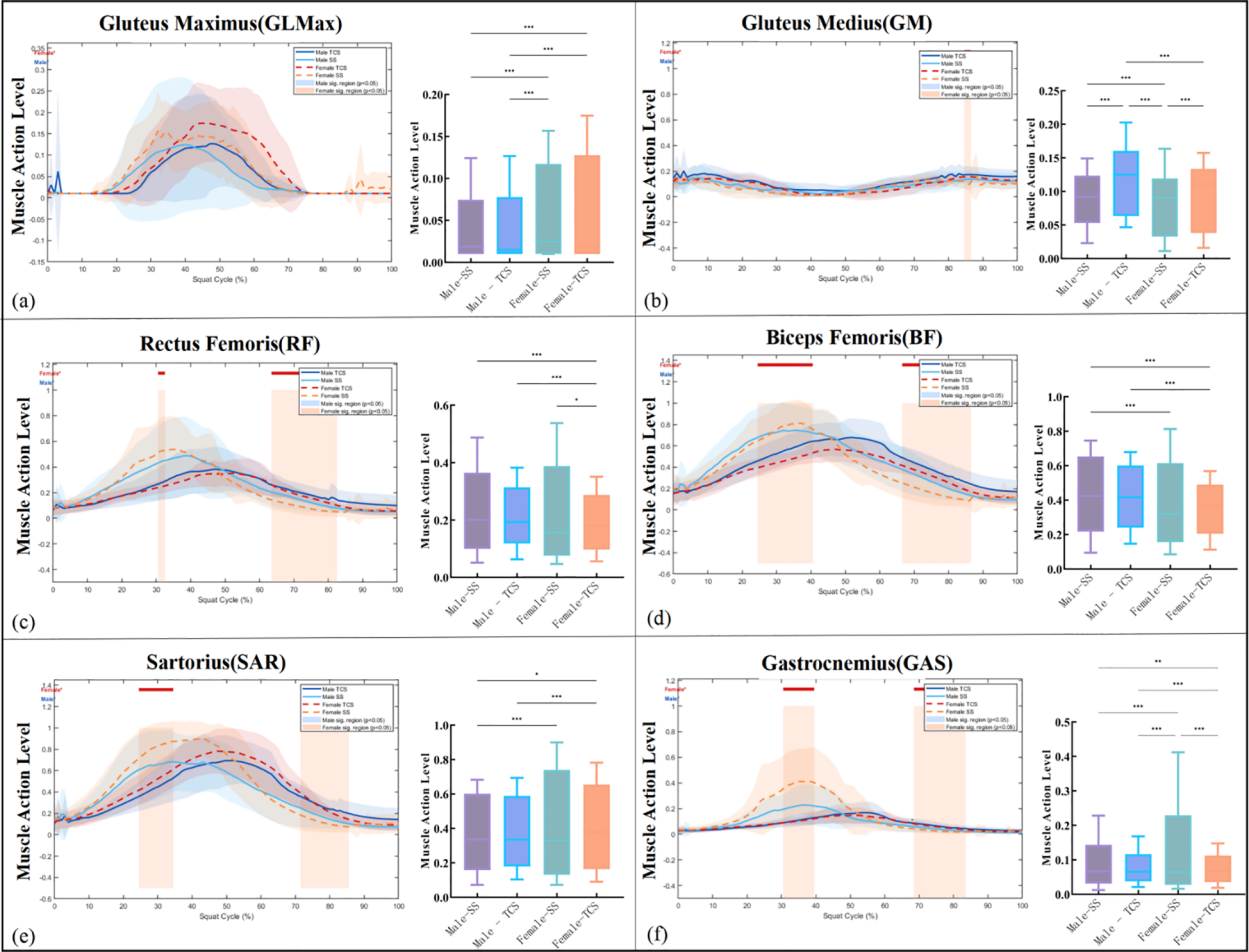
Point-wise and discrete statistical comparisons of lower limb muscle activation patterns between TCS and SS across the full squat cycle. Time-series activation profiles of six lower limb muscles—**(a)** Gluteus Maximus (GLMax), **(b)** Gluteus Medius (GM), **(c)** Rectus Femoris (RF), **(d)** Biceps Femoris (BF), **(e)** Sartorius (SAR), and (f) Gastrocnemius (GAS)—across the complete squat cycle (0–100%) for male (solid lines) and female (dashed lines) participants under TCS and SS conditions.Horizontal bars above each time-series plot indicate time intervals of statistically significant between-condition differences identified by point-wise t-tests (red: female, p < 0.05; blue: male, p < 0.05). Adjacent boxplots present peak activation comparisons across the four groups (Male-SS, Male-TCS, Female-SS, Female-TCS), with significance levels from two-way mixed ANOVA indicated by brackets (*p < 0.05, **p < 0.01, ***p < 0.001).

**Figure 5** presents time-series activation profiles of six lower limb muscles across the full squat cycle. Activation data for RF and BF are derived from directly recorded surface EMG signals; profiles for GLMax, GM, SAR, and GAS represent OpenSim static optimization outputs.

GLMax and GM. GLMax activation exhibited a significant sex × condition interaction. Male participants demonstrated higher peak GLMax activation under SS than TCS, consistent with the greater hip extensor demands of conventional squatting. In contrast, female participants showed higher GLMax activation under TCS than SS, with Female-TCS reaching significantly higher values than all other groups (p < 0.001, **Figures 5A** boxplot). This interaction suggests that female participants recruit the gluteus maximus more extensively during TCS to meet the postural stability demands of the slow, low-stance movement. Point-wise comparisons confirmed sustained significant male-specific differences across approximately 30–60% of the squat cycle (blue bar, **Figure 5A**). For GM, female participants demonstrated greater activation under TCS than SS during the late squat cycle (approximately 80–100%), as indicated by the point-wise significant interval (red bar, **Figure 5B**), potentially reflecting enhanced frontal-plane hip stabilization demands during TCS in females.

RF and BF. RF activation was generally higher under SS in male participants during the mid-cycle knee extension phase, consistent with greater quadriceps demand in conventional squatting. Point-wise comparisons identified significant female-specific differences across the late squat cycle (approximately 60–80%) and a brief male-specific interval during early loading (**Figure 5C**). Conversely, BF activation was markedly elevated under TCS relative to SS, particularly in female participants, reflecting the greater hamstring co-activation demands of the Tai Chi movement paradigm. Sustained female-specific significant intervals were observed across approximately 20–65% of the squat cycle (red bar, **Figure 5D**). Two-way ANOVA confirmed significant main effects of squat type on both RF and BF (p < 0.001), with a significant squat type × sex interaction for BF (p < 0.05), indicating that the shift toward hamstring co-activation during TCS was more pronounced in female participants.

SAR and GAS. SAR activation was substantially higher during TCS in both sexes, with female participants demonstrating particularly elevated recruitment across the mid-to-late squat cycle. Point-wise comparisons confirmed significant female-specific differences spanning approximately 20–50% of the cycle (**Figure 5E**). GAS activation was consistently higher under SS, reflecting greater plantarflexor demands of conventional squatting, with significant female-specific differences identified across a broad mid-cycle region and male-specific differences during the early loading phase (**Figure 5f**). Two-way ANOVA confirmed significant main effects of both squat type and sex on SAR and GAS activation (p < 0.001), with significant interaction effects for both muscles.

Overall pattern. Across all six muscles, TCS was characterized by smoother activation trajectories with relatively higher BF, SAR, and female-specific GLMax recruitment, reflecting a shift toward posterior chain engagement and enhanced knee flexor co-activation. SS elicited more abrupt mid-cycle peaks in RF and GAS, indicating greater demands on knee extensor and ankle plantarflexor musculature. These between-condition differences were consistently more pronounced in female participants, corroborated by the wider point-wise significant time intervals and significant squat type × sex interaction effects observed across multiple muscles.

### 3.4 Biomechanical stress distribution in knee joint structures during different squat types

**Figure 6:**
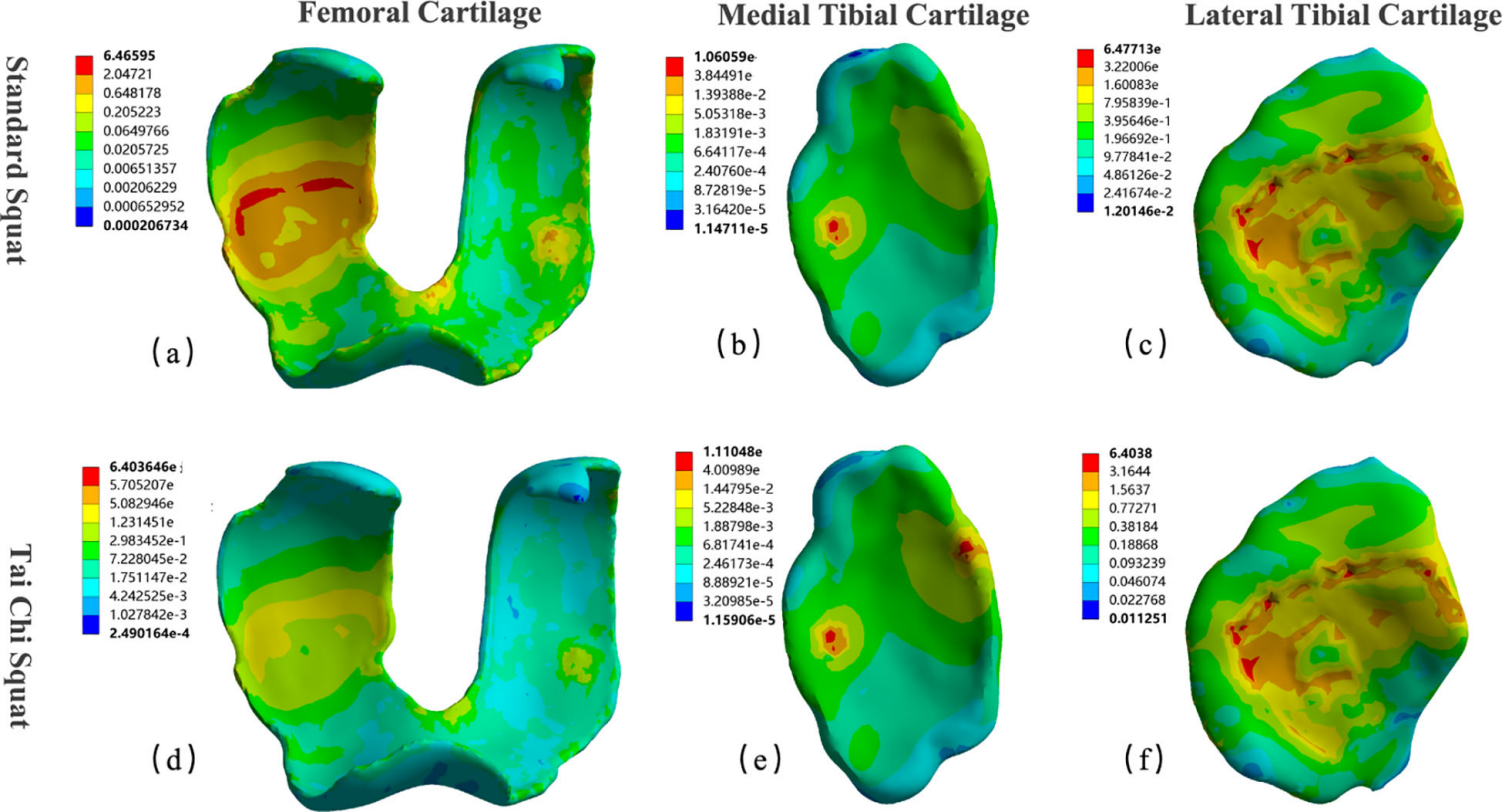
Von Mises stress distribution contours of knee cartilage and menisci under different squatting modes. Stress distributions at the moment of maximum knee flexion are presented for **(a–c)** SS and **(d–f)** TCS. From left to right: femoral articular cartilage, medial tibial cartilage, and lateral tibial cartilage. Color gradients transition from blue (low stress) to red (high stress), with numerical values representing equivalent stress magnitudes across each structure’s surface.

**Figure 6** presents Von Mises stress distribution contour maps of the femoral articular cartilage and menisci at the moment of maximum knee flexion. Color gradients transition from blue (low stress) to red (high stress), with numerical values representing equivalent stress magnitudes across each structure’s surface.

Under both conditions, stress concentration was localized to the posterior femoral condyles, consistent with the contact mechanics expected at large knee flexion angles during deep squatting. Quantitatively, SS generated a peak Von Mises stress of 6.47 MPa on the femoral cartilage, whereas TCS produced a modest reduction to 6.40 MPa. Of greater functional significance, however, were the qualitative differences in stress distribution morphology apparent from the contour maps. Under SS, the stress pattern exhibited a characteristic focal “island” configuration, with steep stress gradients concentrated over a narrow contact region on the posterior condylar surface and sharply demarcated boundaries, indicative of highly localized cartilage pressure. Under TCS, the color band transitions were considerably more gradual, reflecting a more homogeneous distribution of load across the cartilage contact surface. This stress-dispersing effect is mechanically favorable, as it may reduce the risk of localized cartilage fatigue and wear.

The meniscal stress distribution exhibited a pronounced mediolateral asymmetry under both conditions. The lateral tibial cartilage consistently bore substantially greater stress than the medial tibial cartilage, identifying it as the primary load-bearing structure during deep squatting. Relative to SS, TCS reduced peak lateral meniscal stress from 6.48 MPa to 6.40 MPa, and the high-stress zone extended over a slightly broader region of the meniscal body, suggesting that the applied load was distributed across a larger contact area. Regarding the medial tibial cartilage, peak stress under TCS (1.11 MPa) was marginally elevated compared with SS (1.06 MPa); however, these values remained substantially lower than those recorded for the lateral tibial cartilage and femoral cartilage. Furthermore, the corresponding contour maps revealed no pathological stress concentration foci on the medial meniscal surface under either condition.

## 4 Discussion

The present study integrated three-dimensional motion capture, musculoskeletal modeling, and FEA to examine the biomechanical differences between TCS and SS across kinematic, kinetic, and tissue-level stress dimensions. The findings demonstrate that TCS exhibits distinctive adaptive characteristics in lower-limb mechanical patterning.

### 4.1 Mechanisms of joint stability maintenance under elevated knee extensor moments

This study reveals a critical biomechanical paradox: although TCS generated greater peak knee extension moments than SS, particularly in male participants, FEA demonstrated a more homogeneous stress distribution across the femoral cartilage and menisci under TCS. This dissociation between external moment and internal tissue loading indicates that relying solely on the net external joint moment to infer intra-articular injury risk is limited, and that neuromuscular coordination patterns exert a dominant regulatory influence on determining tissue-level load distribution.

The musculoskeletal simulation outputs from OpenSim provide a mechanistic explanation for this observation (Stapornchaisit et al., 2019). The simulated peak muscle forces for the biceps femoris (BF) and sartorius (SAR) were substantially elevated during TCS relative to SS, reflecting a marked shift toward posterior chain co-activation. From a biomechanical standpoint, the quadriceps transmit force via the patellar tendon in a direction that produces anteriorly directed shear at the proximal tibia when contracting at knee flexion angles below 50 degrees, thereby loading the anterior cruciate ligament (ACL) (Escamilla et al., 2012; Taketomi et al., 2024; Lloyd and Buchanan, 2001). The simulated BF forces in this study represent a posteriorly directed counter-force that directly opposes this anterior shear. This protective mechanism is quantitatively supported by Li et al. (1999), who demonstrated that the addition of an antagonist hamstring load significantly reduced ACL *in-situ* forces by 30% to 44% across knee flexion angles of 15 to 60 degrees. Consistent with this, Aagaard et al. (2000) documented that antagonist hamstring moments can counteract up to 75% of the extension moment during slow, controlled knee movements. The elevated BF forces observed in our OpenSim results are therefore mechanistically consistent with a coordinated strategy that attenuates the anterior tibial shear induced by the relatively greater quadriceps demand. The characteristic slow-movement paradigm of TCS appears to be the primary driver for this enhanced antagonist recruitment. Unlike the SS condition, which relies more heavily on passive structural constraints to manage tibiofemoral shear, the slow and continuous weight displacement of TCS compels the central nervous system to recruit antagonist musculature more extensively to maintain postural equilibrium (Liu H et al., 2024). The increased simulated forces in the SAR, a biarticular muscle, further suggest that TCS engages a multi-joint stabilization strategy. This distributed muscular support, captured by the musculoskeletal model, enhances overall joint stiffness and provides a more stable mechanical environment (Baratta et al., 1988). Recent evidence (Azevedo et al., 2025) confirms that high levels of simulated co-activation are essential for tasks requiring the precise motor control characteristic of Tai Chi practice.

Taken together, the simulated muscle force profiles indicate that the elevated knee extension moments under TCS do not translate into greater intra-articular risk. Instead, the high neuromuscular demand, as evidenced by the concurrent increase in agonist and antagonist forces, redistributes the load into an active inter-muscular balance (Slater and Hart, 2017). This redistribution effectively attenuates passive tensile loading on the ACL and promotes the more homogeneous intra-articular stress distribution observed in the FEA results. These findings provide biomechanical evidence that TCS can simultaneously provide a robust training stimulus for the knee extensors while maintaining a protective internal loading environment.

### 4.2 Effect of motion control features on cartilage contact stress distribution

In addition to muscle coordination strategies, the kinematic characteristics of TCS exert an independent effect on articular cartilage stress distribution. OpenSim inverse kinematics analysis indicates that the knee flexion-extension angular velocity curve under TCS conditions is smoother than that of SS, with the peak flexion angle controlled within approximately 80°. In contrast, SS exhibits more abrupt kinetic transitions and greater variance in peak flexion angles. Finite element analysis further reveals that although the absolute difference in peak von Mises stress on the femoral cartilage between TCS and SS is marginal, their stress distribution patterns differ fundamentally. Under SS conditions, stress concentrates in focal high-stress islands on the posterior femoral condyle, accompanied by steep stress gradients. Conversely, TCS demonstrates a more diffuse and uniform stress distribution pattern. From a biomechanical perspective, the slow and uniform motion control characteristic of TCS may improve the cartilage contact mechanics via multiple pathways. First, as a biphasic porous material, the mechanical response of articular cartilage is highly time-dependent. During dynamic loading, interstitial fluid flow and matrix deformation within the cartilage require sufficient time to achieve stress relaxation (Farquhar et al., 1996). The slow execution speed of TCS provides ample time for fluid redistribution within the cartilage matrix. This allows stress to be transmitted uniformly across a broader contact area, thereby preventing the formation of localized high-pressure foci. This contrasts with the findings of Kothurkar et al. (2023). Their study utilized static finite element analysis to simulate deep squats (153° knee flexion), reported exceptionally high peak stresses (19.9 MPa in femoral cartilage), and suggested that sustained deep flexion postures should be avoided to protect knee health. By restricting the peak flexion angle to approximately 80° and reducing the movement rate, TCS effectively avoids the high-stress regions associated with deep flexion.

Second, TCS emphasizes the technical principle of “coherent and circular movement,” requiring practitioners to maintain a smooth center-of-mass shift and a continuous motion trajectory throughout the squat cycle. This controlled movement pattern helps maintain a relatively constant joint contact area and reduces transient stress peaks caused by kinematic impact or sudden loading. A study by Thomeer et al. (2022) using mobile biplane X-ray imaging to assess knee cartilage contact kinematics during daily activities (level walking, stair negotiation, downhill walking) demonstrated that the trajectories of the femoral and patellar contact centers are highly coupled with the tibiofemoral flexion angle. This indicates the deterministic role of joint geometry on contact patterns. The moderate flexion angle range maintained in TCS (approximately 20° to 80°) corresponds precisely to a zone where the tibiofemoral contact area is relatively large and the stress distribution is favorable. Consequently, it avoids the adverse biomechanical environment of deep flexion, where contact area sharply decreases and stress concentration exacerbates (Kothurkar and Lekurwale, 2022). Regarding cartilage injury mechanisms, localized stress concentration, rather than overall load magnitude, is likely the more critical pathogenic factor. Studies have shown that a single applied stress exceeding 25 MPa or cyclic stresses of 5 to 10 MPa can induce cartilage damage (Farquhar et al., 1996; Thambyah et al., 2005). The high-stress island morphology observed under SS conditions indicates the presence of localized stress concentrations, which may induce cartilage micro-cracking and matrix degradation under repetitive loading. In contrast, the diffuse stress distribution pattern of TCS disperses the mechanical load over a larger cartilage contact area, reducing the stress intensity per unit area and potentially delaying the cumulative process of cartilage fatigue damage. This mechanistic explanation is consistent with the improved stress distribution in the lateral meniscus observed in the finite element analysis. The expansion of the high-stress zone and the reduction in peak stress under TCS conditions collectively create a more favorable mechanical environment for cartilage tissue.

In summary, TCS optimizes the internal stress distribution of articular cartilage while maintaining a moderate external training load through its unique motion control features. These features include restricting the peak flexion angle, reducing the rate of angular velocity change, and emphasizing uniform, continuous movement. This biomechanical paradigm of “high load with low injury risk” provides a theoretical basis for the application of Tai Chi in the prevention and rehabilitation of knee osteoarthritis.

### 4.3 Sex differences and neuromuscular adaptation strategies

This study reveals significant sex differences in the muscle activation patterns induced by TCS. OpenSim static optimization results indicate that under TCS conditions, female participants exhibited a significantly greater relative increase in the activation levels ofGLMax, BF, and GM compared to males. Conversely, under SS conditions, male participants demonstrated higher GLMax activation. This finding suggests that TCS may elicit differential neuromuscular training effects between the sexes through distinct mechanisms.

From a biomechanical perspective, female athletes generally exhibit a “quadriceps dominance” strategy during dynamic movements. This indicates they preferentially activate the quadriceps over the hamstrings during landing or deceleration tasks, resulting in a relatively extended and stiff knee posture that increases the risk of ACL injuries (Hughes and Watkins, 2006; Malinzak et al., 2001; Li et al., 2005). Maniar et al. (2022) noted that during landing, quadriceps activation in female athletes is 17% to 40% of maximum voluntary contraction higher than in males, whereas hamstring activation is 20% MVC lower. Huston and Wojtys (1996) further discovered that the initial response to anterior tibial translation in female athletes is quadriceps contraction, while male athletes preferentially contract their hamstrings. This divergence in neuromuscular control strategies, compounded by a larger Q-angle in females (averaging 13.9° versus 11.5° in males) and higher dynamic knee valgus angles, collectively establishes the biomechanical basis for female ACL injury rates being two to eight times higher than those of males (Hughes and Watkins, 2006; Hewett et al., 2005). The significant enhancement of posterior chain muscle (GLMax, BF, and GM) activation observed in females under TCS conditions in this study indicates that the slow and controlled motion paradigm of Tai Chi may effectively correct quadriceps dominance. Mechanistically, enhanced co-activation of the gluteus maximus and hamstrings can improve the biomechanical environment of the knee joint through several pathways. First, stronger gluteal activation helps maintain frontal plane pelvic stability and reduces the dynamic knee valgus angle (Khayambashi et al., 2016). Second, the posterior shear force generated by hamstring contraction counteracts the anterior tibial translation induced by the quadriceps, thereby decreasing ACL tension (Li et al., 1999). Third, the co-contraction of posterior chain muscles increases overall joint stiffness and enhances dynamic stability (Granata et al., 2002).

Notably, research by Granata et al. (2002) demonstrated that quadriceps and hamstring stiffness in females across all torque levels is only 55.8% to 73.9% of that in males, which may partially explain the higher susceptibility of females to ACL injuries. The enhanced posterior chain muscle activation induced by TCS in females in the present study may represent a functional compensatory mechanism. By increasing muscle activation levels to offset relatively lower muscle stiffness, females can achieve joint stability comparable to or even better than that of males. In contrast, the higher GLMax activation exhibited by male participants under SS conditions likely reflects their existing ability to adequately mobilize the posterior chain musculature during standard squats. However, TCS did not elicit a relative increase in males similar to that observed in females. This may be because the baseline posterior chain activation level in males is already high, leaving limited room for further enhancement. This finding regarding sex differences aligns with the results of Smith et al. (2020; 2021). Their study found that the level of muscle co-contraction during weight-bearing tasks was significantly higher in females than in males, suggesting that women rely more heavily on neuromuscular strategies to compensate for inherent anatomical and mechanical disadvantages. From the perspective of preventing knee osteoarthritis (OA), the sex differences identified in this study hold substantial clinical significance. Females represent a high-risk population for knee OA, with risk factors including lower lower-extremity muscle strength, greater knee valgus angles, and more pronounced asymmetrical joint loading (Powers, 2010). By promoting greater recruitment of the posterior chain muscles in women, TCS may help improve load distribution across the knee joint and reduce localized stress concentrations in the cartilage, thereby lowering the risk of OA development. This mechanism resonates with the ACL injury prevention strategies for females proposed by Mancino et al. (2024). They suggested utilizing neuromuscular training to enhance posterior chain strength and coordination as a method to improve the dynamic stability of the knee.

In summary, the findings of this study demonstrate that TCS exerts differential neuromuscular training effects on both sexes. For females, TCS may improve the knee biomechanical environment by correcting quadriceps dominance and enhancing posterior chain activation. For males, TCS maintains and optimizes existing muscle coordination patterns. This sex-specific adaptation mechanism provides a theoretical foundation for the personalized application of Tai Chi in knee joint injury prevention and rehabilitation.

### 4.4 Applied implications and study limitations

By integrating motion capture, musculoskeletal simulation, and finite element analysis, this study elucidated the underlying biomechanical advantages of TCS compared to the SS. The slow and controlled movement paradigm of TCS optimizes intra-articular stress distribution and enhances posterior chain co-activation. This provides a highly efficient rehabilitation alternative for joint preservation and offers a targeted neuromuscular strategy to correct quadriceps dominance in female athletes for ACL injury prevention, thereby scientifically validating the clinical utility of traditional Tai Chi principles. To build upon these cross-sectional findings, future research must prioritize longitudinal randomized controlled trials and targeted clinical interventions among high-risk populations, such as older adults and individuals with knee osteoarthritis or post-ACL reconstruction. Concurrently, advanced biomechanical investigations should develop subject-specific models utilizing magnetic resonance imaging, incorporate time-varying dynamic loads, and evaluate the complete lower extremity kinematic chain to precisely quantify the long-term therapeutic efficacy and multi-joint coordination strategies associated with TCS.

The present study has several limitations that should be acknowledged. First, the age discrepancy between male (41.3 ± 8.8 years) and female (54.3 ± 3.2 years) participants may confound sex-specific interpretations, as age-related differences in neuromuscular function and cartilage properties cannot be fully excluded; future studies should recruit age-matched cohorts. Second, the FEA component employed a single generic male knee model derived from the Visible Human Project, with a chosen element size of 1.5 mm justified by reference to published convergence data from comparable models rather than an independently conducted mesh convergence analysis; subject- and sex-specific anatomical variations in bone geometry, cartilage thickness, and ligament morphology were therefore not captured, limiting the direct applicability of absolute stress estimates. Third, loading conditions were represented by static peak joint moments and muscle forces applied at the femoral head center, rather than dynamic time-varying loads across the full squat cycle, which may underestimate inertial and viscoelastic contributions to transient stress peaks. Fourth, all biological tissues were modeled as isotropic linear elastic materials; cartilage and ligaments exhibit anisotropic, nonlinear, and viscoelastic behavior *in vivo*, and future models should incorporate more biofidelic constitutive frameworks. Fifth, point-wise t-tests were employed to characterize temporal differences in continuous waveform data, as individual-level time-series data were not available for re-analysis, precluding formal 1D-SPM, which would provide more rigorous Type I error control across the temporal domain; future work should archive subject-specific waveform data to enable its implementation. Furthermore, model validation was limited to two muscles (RF and BF) using Pearson correlation coefficients alone, While the correlation values obtained (r = 0.840 and r = 0.779) are consistent with accepted thresholds in the musculoskeletal modeling literature, future studies should employ intramuscular EMG or ultrasound-guided recording to enable more comprehensive multi-muscle validation.

## 5 Conclusion

This study demonstrates that TCS confers measurable biomechanical advantages over SS through mechanisms distinct from external moment reduction. Despite generating greater peak knee extension moments than SS, particularly in male participants, TCS promoted more controlled knee flexion trajectories and more balanced posterior chain co-activation patterns, collectively optimizing internal load distribution across the knee. Finite element analysis further revealed a more homogeneous stress distribution within the femoral cartilage and menisci during TCS, suggesting a protective effect against localized cartilage fatigue. The findings also provide preliminary evidence that female participants may exhibit relatively greater neuromuscular adaptations to TCS, particularly in terms of enhanced posterior chain musculature recruitment. Collectively, these findings provide preliminary biomechanical evidence supporting the potential application of TCS in knee injury prevention and rehabilitation program design.

## Data Availability

The raw data supporting the conclusions of this article will be made available by the authors, without undue reservation.
